# KmPred: prediction of Michaelis constants (Km) using an integrative machine learning framework

**DOI:** 10.3389/frai.2026.1711471

**Published:** 2026-01-30

**Authors:** Meshari Alazmi

**Affiliations:** College of Computer Science and Engineering, University of Ha’il, Ha’il, Saudi Arabia

**Keywords:** bioinformatics, drug discovery, KmPred, metabolic engineering, Michaelis–Menten constant (Km), molecular descriptors, protein embeddings, systems biology

## Abstract

**Background and motivation:**

The Michaelis constant Km is one of the key kinetic parameters in the quantification of enzyme-substrate affinity within the context of the Michaelis–Menten theory. While Km values are traditionally subjected to labor-intensive governance via *in vitro* assays, the brisk expansion of protein sequence and chemical databases has composed an essential intended for computational prediction approaches.

**Methodology:**

Herein, we expose a consolidative machine learning framework-KmPred-for Km forecast that merges protein sequence embeddings from state-of-the-art language models with molecular descriptors derived from substrate SMILES descriptions. This methodology was benchmarked on the MPEK dataset and the independent dataset assembled by Kroll et al.

**Results and discussion:**

On the MPEK dataset, the greatest model achieved a test MSE of 0.4995, RMSE of 0.7067, MAE of 0.5022, R^2^ of 0.7049, and a PCC of 0.8398 (*p* < 1 × 10−6), outperforming the baseline MPEK model. On the Kroll dataset, KmPred achieved a test MSE of 0.6206, RMSE of 0.7878, R^2^ of 0.5519, PCC of 0.7440, and Spearman’s ρ of 0.7342, which represents reasonable results compared to state-of-the-art methods. These outcomes demonstrate that combining multi-modal protein sequence and ligand features with advanced machine learning architectures enables robust and generalizable Km prediction across diverse datasets. Specifically, we utilized LSTM and Transformer models solely for feature extraction to capture complex sequential and contextual patterns from enzyme sequences, while employing XGBoost as our primary regression model for final Km predictions. Beyond methodological impact, this work highlights the role of AI-driven kinetic modeling in accelerating enzyme characterization, facilitating metabolic engineering, and enhancing drug discovery pipelines. Our approach thus establishes a foundation for predictive enzymology at scale, with significant potential to benefit biotechnology, synthetic biology, and national strategic initiatives such as Saudi Vision 2030.

## Introduction

Enzymes are necessary biological catalysts, transforming, in effect, all biochemical pathways-from central carbon metabolism down to detailed biosynthetic pathways-through letting down of activation energy into physiologically applicable periods and giving exquisite control over cellular metabolism. One of the desperate parameters that outline enzyme activity is the Michaelis–Menten constant, Km, which designates the substratum intensity by which an enzyme exerts semi of its maximum catalytic velocity, Vmax (Michaelis and Menten, 1913). Within the frame of the Michaelis–Menten equation, Km acts as a converse quantity of substrate affinity: enzymes with low Km values reach high catalytic competence at low substrate attentions, while high Km values indicate weaker binding (Cornish-Bowden, 2013). The precise determination of Km is central to many characteristics of the life sciences. In metabolic engineering, Km values are needed to tune enzyme usage in artificial trails and to improve flux supplies in bioproduction (Nielsen and Keasling, 2016). In drug discovery, Km is important for the analysis of enzyme-inhibitor interactions, especially for aggressive inhibitors, which change the substrate affinity (Copeland, 2013). In clinical research, reassessed Km values of mutant enzymes are often associated with metabolic disorders, rendering Km a critical diagnostic and therapeutic marker (Srinivasan, 2023; Segel, 1975). Traditionally, Km has been determined via *in vitro* kinetic assays, commonly based on spectrophotometric, fluorometric, or chromatographic techniques. While accurate, these assays are resource-intensive, require purified enzymes and substrates, and do not scale well to the now rapidly expanding universe of newly sequenced proteins and chemical entities.

The exponential growth of omics databases has made it possible to investigate enzyme kinetics in depth on extraordinary scales. Numerous protein sequences have been produced by high-throughput sequencing advancements, and rich molecular libraries are available through cheminformatics sources. In order to lessen reliance on laboratory kinetics, there is an urgent need for computational techniques that can predict Km entirely from arrangement and molecular information. By incorporating kinetic parameters at the genome scale, these predictive models would advance schemes biology models, maintain tailored medicine by providing additional estimates for disease-associated variations, and speed up enzyme engineering.

The analysis of biological data is changing as a result of these recent advances in machine learning and artificial intelligence. Transformer-based designs are driven by biological protein linguistic models like ProtBERT (Elnaggar et al., 2021), UniRep (Alley et al., 2019), and ESM-2 (Lin et al., 2023) to extract structural and functional features from raw arrangements. These models interpret underlying biochemical messages without the need for specific structural input. On the chemical side, substrate development and adaptability are effectively encoded by SMILES-based representations (Weininger, 1988), molecular descriptors, and fingerprints like ECFP and MACCS-Keys (Rogers and Hahn, 2010; Durant et al., 2002). This addition of information about proteins and small molecules has further paved the way for the use of multimodal learning frameworks in forecasting. Pioneering landmark works demonstrated that ML can predict enzyme kinetic parameters. Heckmann et al. (2018) conducted a ML model to study the prediction of turnover numbers across a wide variety of enzymes, while Lai and Xu (2022) modeled Km with deep learning and obtained reasonable accuracy with the use of sequence and structural features. Notwithstanding these developments, difficult challenges remain on how to avoid overfitting with high-dimensional features, how to integrate dissimilar biochemical forms into a unified prognostic model, and particularly how to attain vigorous simplification across an extensive variety of enzyme families and chemical constructions.

**Figure 1:**
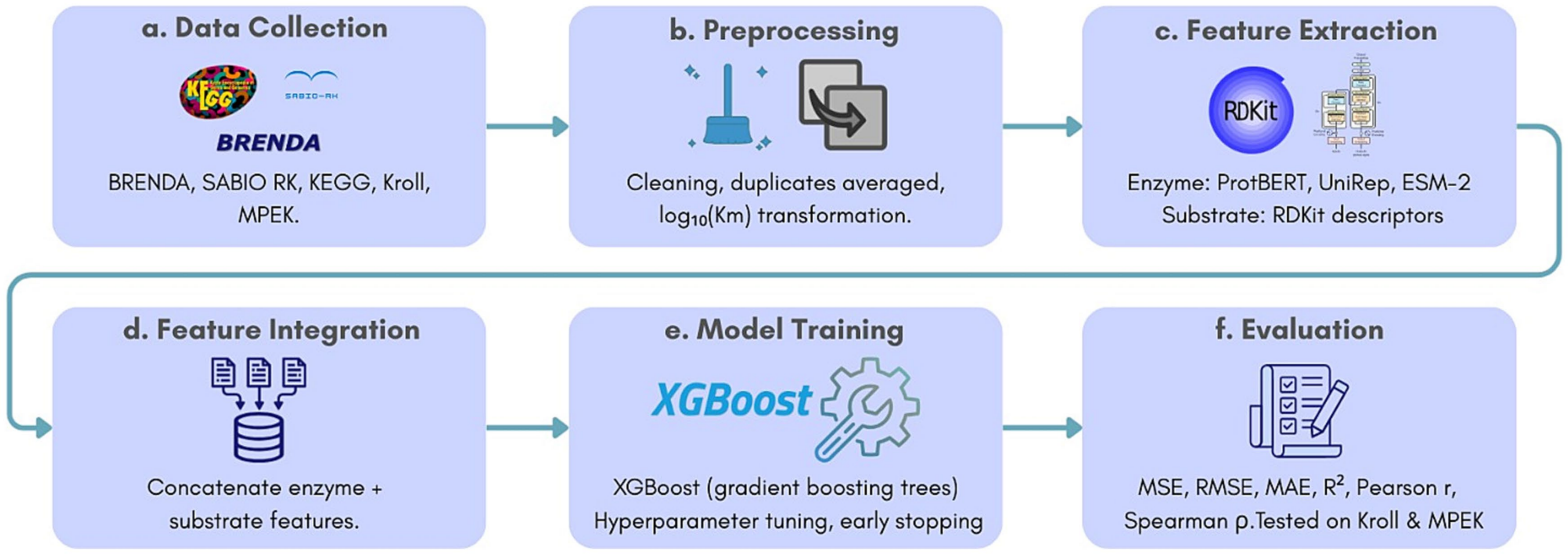
Overview of the KmPred framework.

In this work, we propose a comprehensive machine learning framework to directly predict Km values from enzyme sequences and substrate structures. Our approach is summarized in Figure 1, incorporating three key components: (1) protein embeddings generated using state-of-the-art language models including LSTM-based (UniRep) and Transformer-based architectures (ESM-2, ESM1b, ProtBERT, ProtT5) for feature extraction, (2) molecular descriptors extracted via cheminformatics tools, and (3) XGBoost as our primary regression model for Km prediction. We conduct a comprehensive evaluation by systematically benchmarking our approach against baseline models, demonstrating that deep learning-derived features combined with gradient boosting regression achieve superior predictive performance. The importance of this research work is related to overcoming computational challenges not only in enzyme kinetics but also, in general, to applications in biotechnology and drug discovery for the development of the bioeconomy in view of Saudi Vision 2030 (Kingdom of Saudi Arabia, 2016).

## Methodology

### Data collection and preprocessing

Kinetic data for enzyme-substrate interactions were collected from three open-access enzymology databases: BRENDA (Chang et al., 2021), SABIO-RK (Wittig et al., 2018), and KEGG (Kanehisa et al., 2021).

The Km values were transformed into their logarithmic form, log10 (Km), for numerical stability and comparability over the wide range of scales observed. The next crucial step in cleaning up information was to eliminate entries that lacked either the substrate’s canonical SMILES notation or the enzyme sequence of amino acids in order to preserve data reliability. Additionally, in order to prevent data from becoming redundant and guarantee that the final dataset will be reliable and of superior quality for modeling purposes, the average of the tests was computed and kept for enzyme-substrate pairs that included several Km values.

### Dataset summary

In order to evaluate KmPred’s performance and generalizability more rigorously, two benchmark datasets were used that are quite different in size, curation, and partitioning strategy. For Kroll et al., the dataset consists of 11,675 Km values, split into 80% for training/validation and 20% for independent testing (Kroll et al., 2021). The larger MPEK dataset is composed of 24,585 Km values and uses a 90% train/validation split and 10% independent test set (Wang et al., 2024). In both, all K_m values were log₁₀-transformed to reduce the effect of skewed distributions and ensure numerical stability during regression modeling. All these key details-size, partitioning, and preprocessing-are shown in Table 1.

**Table 1:** Summary of datasets used in this study.

Dataset	Total Km values	Train+ validation	Test	Average Km	Unique enzymes	Unique substrates	Mutant/wildtype ratio
Kroll et al.	11,675	80%	20%	−0.766	6,987	1,496	0/100%
MPEK	24,585	90%	10%	−0.716	8,603	3,082	31/69%

All Km values were log₁₀-transformed for modeling.

### Feature extraction

Several cutting-edge protein language models, such as ProtBERT (Elnaggar et al., 2021), UniRep (Alley et al., 2019), and ESM-2 (Lin et al., 2023), have been used to generate amino acid sequences. The structural and contextual biochemical data contained in the amino acid patterns was captured by these embeddings. Additionally, the iFeatureOmega platform (Chen et al., 2022) was used to generate manually produced descriptors that included secondary-structure-related indices, physicochemical properties, and amino acid composition. The substrate molecules were preprocessed using RDKit (Landrum, 2016) and represented using their canonical SMILES strings (Weininger, 1988). ECFP (Rogers and Hahn, 2010), MACCS keys (Durant et al., 2002), fundamental indicators such as atom counts and bond types, and topological and physicochemical indices (Todeschini and Consonni, 2009) are among the classes of molecular signifiers that were produced.

### Feature integration

The protein embeddings and molecular descriptors for each enzyme and its substrate combination were combined into one vector of features in order to prepare the model. The models were able to together identify and gain insight into the fundamental variables of catalytic efficiency as well as the biochemical variables that influence substrate recognition thanks to one visual representation.

### Model building

Three groups were randomly selected from the MPEK dataset: 10% for validation, 10% for an independent testing set, and 80% for training. However, the Kroll et al. dataset had a different division: 80% for combined training and validation and 20% for the independent testing set. For feature extraction, we employed advanced protein language models including LSTM-based architectures (UniRep) (Hochreiter and Schmidhuber, 1997) and Transformer-based models (ESM-2, ESM1b, ProtBERT, and ProtT5) (Rives et al., 2021) to generate high-dimensional embeddings from enzyme sequences. These deep learning models effectively captured sequential dependencies and contextual information within the protein features. The extracted embeddings, combined with ligand descriptors, were then used as input features for our main predictive model: XGBoost (Chen, 2016). We fine-tuned the XGBoost regressor and reported the final hyperparameters in Table 2. All model code was implemented in Python, utilizing libraries such as scikit-learn (Pedregosa et al., 2011), PyTorch (Paszke et al., 2019), and XGBoost (Chen, 2016). Model hyperparameters were optimized on the validation set, and practices such as early stopping were employed to prevent overfitting.

**Table 2:** Summary of XGBoost hyperparameters.

Hyperparameter	Kroll’s dataset	MPEK dataset	Description
Objective	Regression: Squared Error	Regression: Squared Error	Loss function for optimization (minimizes MSE for regression)
N_estimators/num_boost_round	2500 (fixed)	5000 (max with early stopping)	Number of trees to build; MPEK stops early if validation does not improve
Learning_rate / eta	0.1	0.03	Step size for each tree; lower values = slower, more conservative learning
Max_depth	8	8	Maximum tree depth; deeper trees capture more complexity but may overfit
Subsample	1	0.8	Fraction of samples used per tree; <1.0 adds randomness to prevent overfitting
Colsample_bytree	1	0.8	Fraction of features used per tree; <1.0 reduces tree correlation
Reg_alpha (L1)	0.9	0.0	L1 regularization; pushes weights to zero for feature sparsity
Reg_lambda (L2)	10.0	2.0	L2 regularization; penalizes large weights to smooth the model
Min_child_weight	1	1	Minimum weight required to create a child node; prevents splitting on small samples
Tree_method	‘hist’	‘hist’	Tree building algorithm; ‘hist’ uses histograms for fast, approximate splits
Device	‘cuda’	‘cuda’	Computing device; ‘cuda’ uses GPU for faster training
Random_state/seed	42	42	Random seed for reproducible results

### Model tuning and final parameters

The XGBoost models for both datasets were carefully tuned to balance predictive performance with computational efficiency and generalization capability. Both models utilized the squared error objective function with the histogram-based tree building method (‘hist’) on GPU hardware (‘cuda’) to accelerate training, while maintaining reproducibility through a fixed random seed (42). A shared maximum tree depth of 8 was employed to capture complex non-linear relationships without excessive overfitting. The key distinction in hyperparameter strategies reflects the differing characteristics of the datasets: the Kroll model employed a more aggressive regularization approach with higher L1 (alpha = 0.9) and L2 (lambda = 10.0) penalties to handle its larger feature space and prevent overfitting, while using full subsampling (subsample = 1.0, colsample_bytree = 1.0) and a moderate learning rate (0.1) with a fixed 2,500 trees. In contrast, the MPEK model adopted a more conservative learning strategy with a lower learning rate (0.03) and implemented stochastic training through reduced subsampling ratios (0.8 for both samples and features), which introduces beneficial randomness to improve generalization. The MPEK configuration also employed early stopping with up to 5,000 boosting rounds and lighter regularization (alpha = 0.0, lambda = 2.0), allowing the model to fully leverage the dataset’s informative features while the early stopping mechanism automatically prevented overfitting. This dataset-specific tuning approach proved highly effective, as evidenced by the strong performance metrics and minimal standard deviations across cross-validation folds for both datasets.

### Model evaluation and experimental workflow

The model evaluation was conducted using a set of complementary metrics applied to an independent test set to ensure a comprehensive and practical assessment of predictive performance. Prediction accuracy was primarily quantified using the root mean squared error (RMSE). In addition, the Pearson correlation coefficient was used to assess the linear agreement between predicted and experimentally measured values, while the Spearman rank correlation coefficient was employed to evaluate the monotonic relationship between predictions and ground-truth measurements, independent of linearity assumptions. The coefficient of determination (R2) was further reported to indicate the proportion of variance in the experimental data explained by the models. To assess the statistical robustness and stability of the results, cross-validation was performed across multiple random splits of the dataset. The entire evaluation workflow was designed to be fully reproducible and organized into three distinct stages: feature generation, feature integration, and model training and evaluation.

### Cross-validation set-up

To rigorously assess model performance and ensure generalizability, we implemented a 5-fold cross-validation framework on both the Kroll and MPEK datasets shown in Table 3. For the Kroll dataset, consisting of 11,696 samples with 3,952 features each, we first reserved 2,342 samples (20%) as a held-out test set to provide an unbiased final evaluation. The remaining 9,354 training samples were then divided into five folds, where each fold served as a validation set (approximately 1,870–1,871 samples) while the other four folds were used for training (approximately 7,483–7,484 samples). This approach yielded five independent models, each evaluated on both its respective validation fold and the held-out test set. Feature selection was performed independently within each fold, selecting the top 6,000 most informative features (3,000 from ligand features and 3,000 from sequence features) using univariate regression scoring to prevent information leakage from validation data into the training process. By training on different data subsets and averaging performance metrics across folds, this methodology provides robust estimates of model performance while minimizing overfitting and reducing the influence of any single data split. The consistency of results across folds, measured by standard deviations, serves as a strong indicator of model stability and reliability.

**Table 3:** The average performance of 5-folds cross validation of KmPred tool.

Dataset	MSE (±SD)	R²(±SD)	Pearson(± SD)	Spearman(± SD)
Kroll’s validation set	0.71 ± 0.026	0.49 ± 0.02	0.70 ± 0.013	0.69 ± 0.016
Kroll’s testing set	0.67 ± 0.013	0.51 ± 0.009	0.72 ± 0.006	0.71 ± 0.006
MPEK validation set	0.52 ± 0.009	0.69 ± 0.004	0.83 ± 0.002	0.82 ± 0.005
MPEK testing set	0.51 ± 0.01	0.70± 0.006	0.84 ± 0.003	0.84 ± 0.002

## Results and discussion

Baseline results were adopted from the original publications and were not retrained in this work. For the Kroll dataset, evaluation was performed using the identical test set released by Kroll et al. via their official GitHub repository, enabling direct comparison on the same samples. For the MPEK dataset, results were taken from the original study, which employed a data split of 10% for testing, 10% for validation, and the remaining data for training.

### Performance on Kroll’s dataset

**Figure 2:**
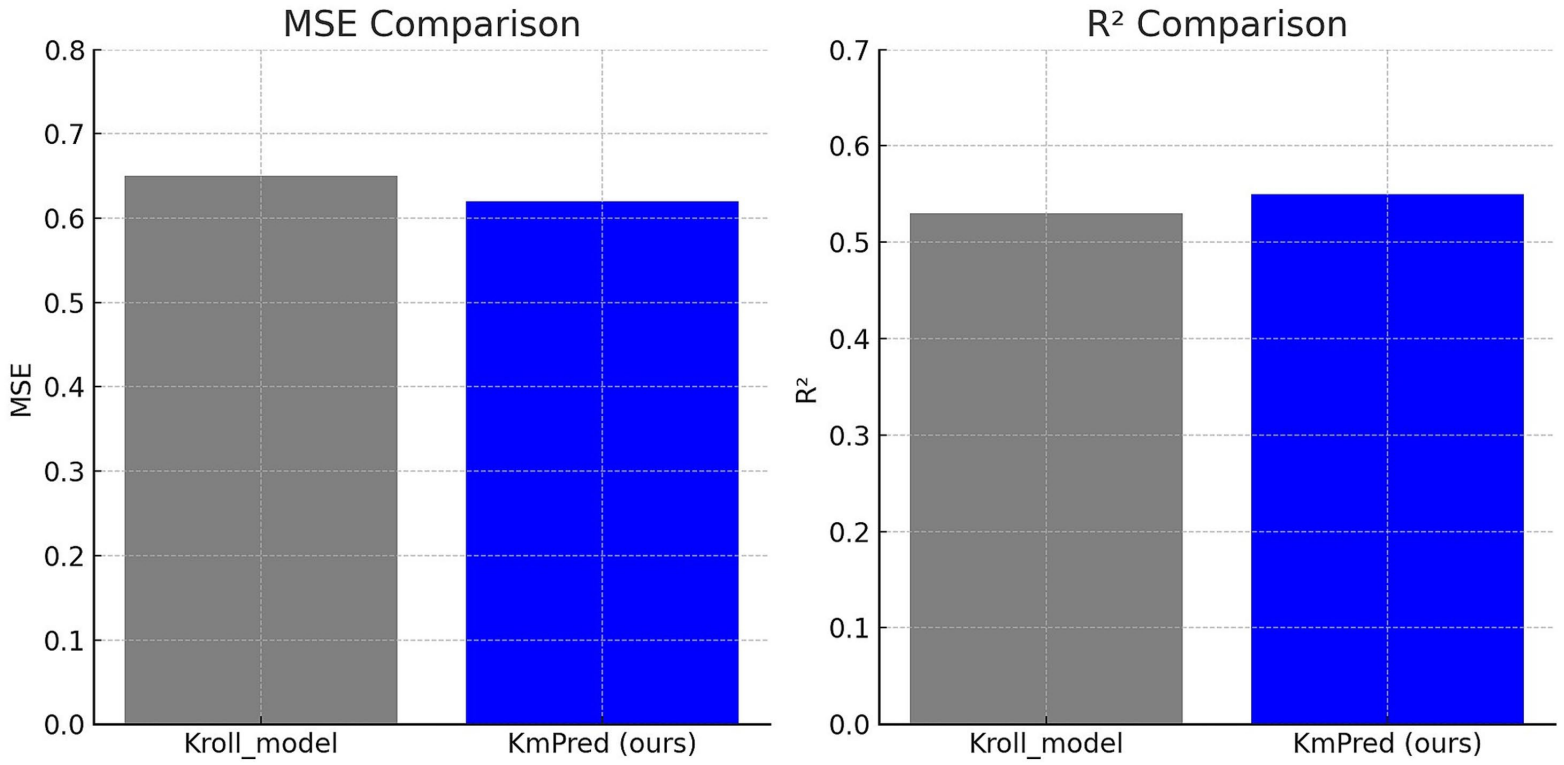
Performance comparison: Kroll_model and KmPred on the Kroll dataset (MSE and R^2^ values).

The predictive capacity of KmPred was initially benchmarked against the Kroll baseline model on the Kroll dataset. As shown in Table 4, KmPred attained a mean squared error (MSE) of 0.62 and a coefficient of determination (R^2^) of 0.55, evaluated to the Kroll baseline which yielded MSE = 0.65 and R^2^ = 0.53. Although the absolute differences may appear modest, the enhancement is expressive in the context of enzyme–substrate affinity prediction, where supplementary variance supported denotes competent acquire of the core biochemical signal. Figure 2 visualizes this comparative analysis, clearly demonstrating KmPred’s superior performance through its lower error and higher explained variance.

**Table 4:** Performance of KmPred and Kroll’s model on the Kroll dataset (test set).

Method	MSE	R^2^
Kroll model	0.65	0.53
KmPred (ours)	0.62	0.55

### Performance on Kroll’s dataset on identical or homologous sequences

As shown in Table 5, our model demonstrates robust predictive performance across different levels of sequence similarity between training and test sets, with MSE values that appropriately reflect the difficulty of each prediction task. When evaluated on the complete test set of 2,342 samples without removing any sequences, the model achieves an MSE of 0.62, establishing a strong baseline performance. When we remove identical sequences (100% similarity) from the test set, reducing it slightly to 2,292 samples, the MSE increases modestly to 0.76, indicating that the model relies minimally on memorizing exact training sequences and generalizes well to novel but similar enzymes. This trend continues when removing sequences with 90 and 50% homology (MSE of 0.75 and 0.76, respectively, both with ~2,292 test samples). Notably, the test set size remains nearly identical across these two similarity thresholds (2,292 and 2,293 samples), indicating that very few sequences fall in the 50–90% similarity range. The minimal change in MSE between these conditions (0.75 vs. 0.76) despite the stricter similarity cutoff demonstrates that the model maintains remarkably consistent performance even when tested on enzymes with moderate sequence divergence from the training data, and that its predictions do not rely heavily on high similarity matches. The most challenging scenario occurs when removing all sequences with more than 10% homology, which drastically reduces the test set to only 41 highly divergent samples and increases MSE to 1.38. This substantial increase is expected because the dramatically smaller test set (98% reduction in size) represents extremely dissimilar enzymes that share minimal sequence features with the training data. Importantly, even in this most stringent evaluation, the model still achieves meaningful predictions (Pearson correlation of 0.58), demonstrating its ability to capture fundamental enzyme-substrate relationships beyond simple sequence similarity. Overall, these results validate that our model generalizes effectively across a wide range of sequence similarities, with performance degradation proportional to both the biological difficulty of the task and the reduced statistical power from smaller test sets.

**Table 5:** Performance of KmPred on the test set based on sequence similarity.

Identical or homologous percentage	MSE	R^2^	Pearson correlation	Spearman correlation	Test set size
No removal of any test samples	0.62	0.55	0.74	0.73	2342
There are no identical sequences (100%) shared between training and testing datasets.	0.76	0.47	0.69	0.68	2292
There are no homologous sequences more than 90% shared between training and testing.	0.75	0.47	0.69	0.68	2292
There are no homologous sequences more than 50% shared between training and testing.	0.76	0.47	0.68	0.68	2293
There are no homologous sequences more than 10% shared between training and testing.	1.38	0.26	0.58	0.59	41

### Performance on the MPEK dataset

**Figure 3:**
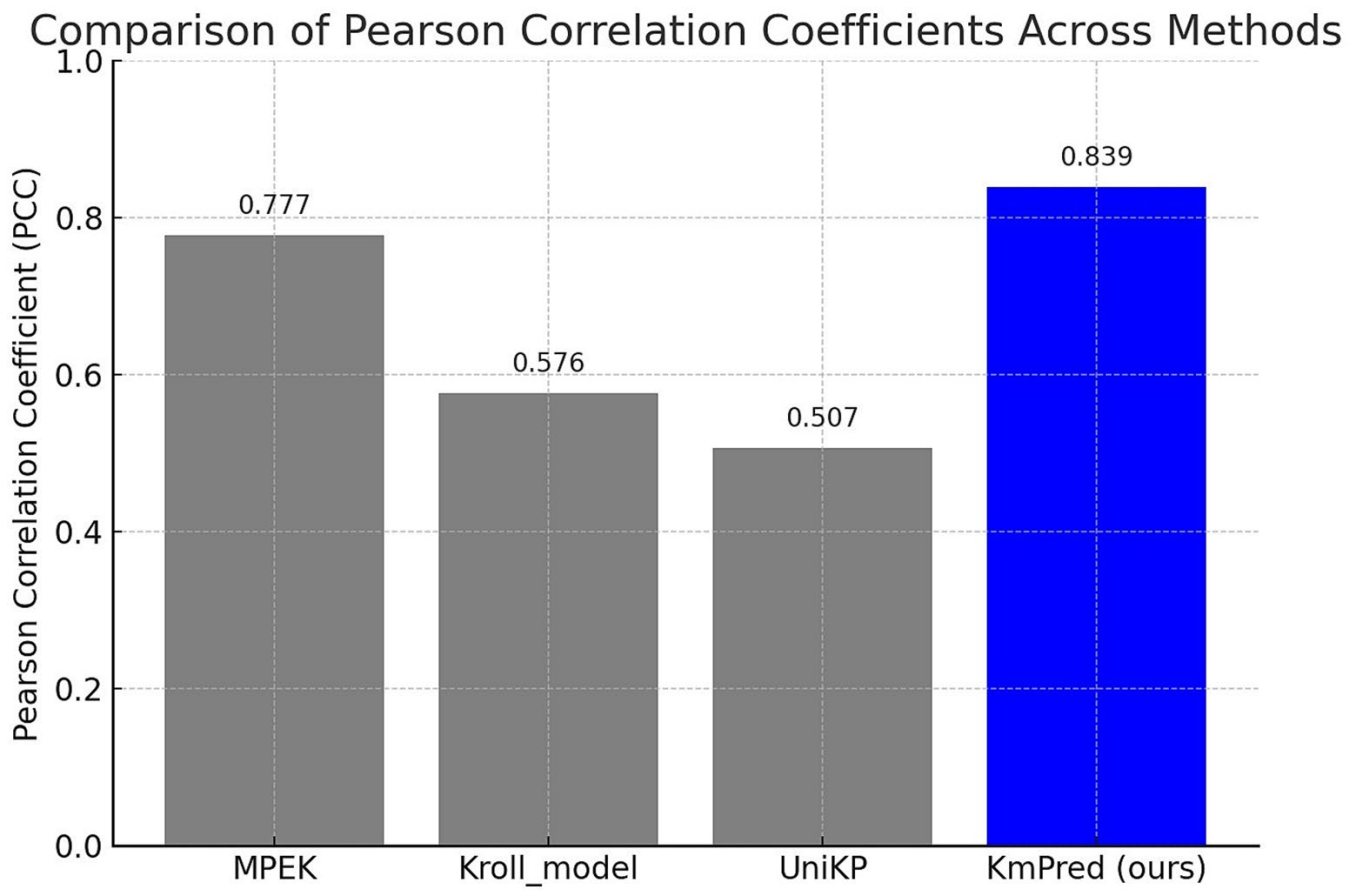
Bar chart: comparison of Pearson correlation coefficient (PCC) across methods on the MPEK dataset.

KmPred demonstrated superior predictive power on the MPEK dataset, achieving a Pearson correlation coefficient (PCC) of 0.839. This value considerably outperforms the standard methods: MPEK with 0.777, Kroll_model with 0.576, and UniKP with 0.507. These comparative outcomes, also visually indicated in Figure 3, finally start KmPred as the most dependable predictor among the judged attempts on this extensive standard. Table 6 obviously demonstrates that KmPred is far greater, with a PCC of 0.839, as related to the other benchmark approaches on the MPEK dataset.

**Table 6:** Pearson correlation coefficient (PCC) comparison of KmPred against other methods on the MPEK dataset.

Method	PCC
MPEK	0.777
Kroll_model	0.576
UniKP	0.507
KmPred (ours)	0.839

### Predicted vs. actual log₁₀(Km) scatter plots

**Figure 4:**
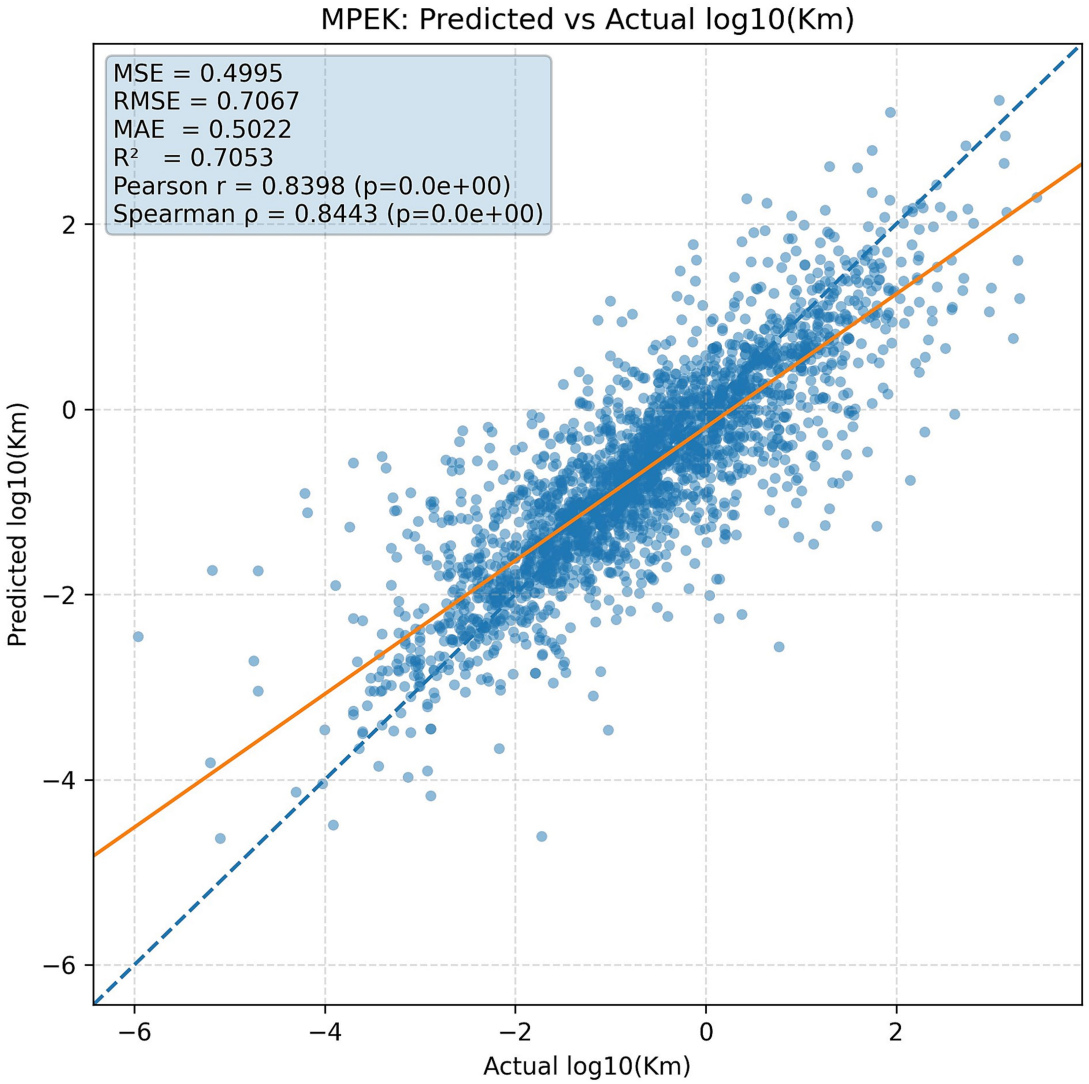
Predicted vs. actual log₁₀(K_m_) on the Kroll dataset.

**Figure 5:**
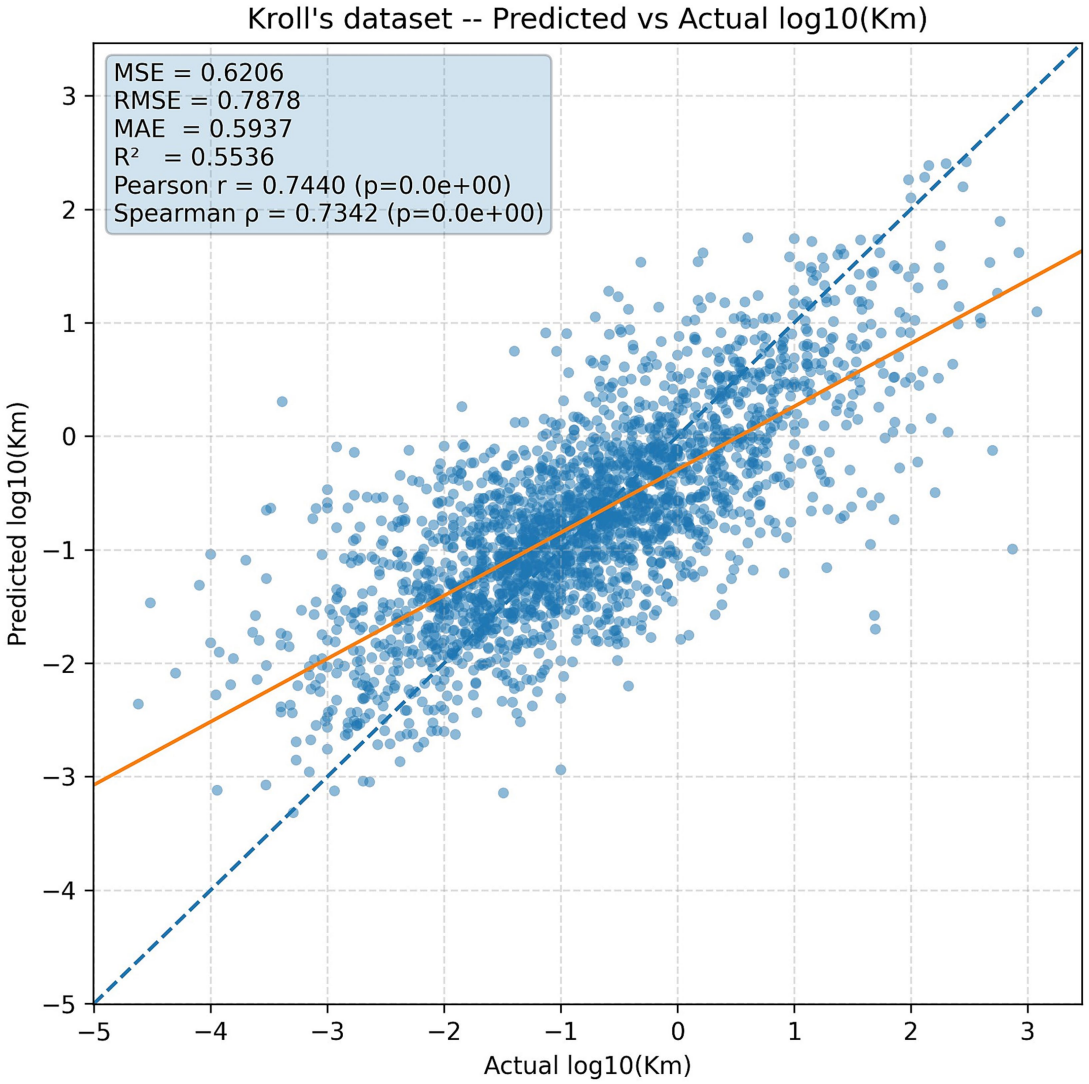
Predicted vs. actual log₁₀(K_m_) values on the MPEK dataset.

To check the quality of KmPred forecasts, we observed scatter plots of forecast versus experimentally measured log₁₀(Km) values. As Figure 4 for the Kroll dataset illustrates, the predictions clustered strongly around the identity line, y = x, in concordance with the obtained PCC of 0.744. For the MPEK dataset, the alignment is even tighter (Figure 5), reflecting a significantly higher PCC of 0.839 and hence showing KmPred’s improved predictive fidelity even when challenged with broader data diversity. The figure’s dashed red line represents the identity line y = x, where the points of perfectly predicted values should fall.

This plot thus illustrates that the data points all lie very near to the identity line y = x, hence visually confirming the attainment of high predictive accuracy on the MPEK dataset.

## Discussion

A key limitation of the present study, shared with prior large-scale Km prediction efforts, is the absence of detailed assay condition metadata associated with the reported Km values. Experimental Km measurements are known to depend on factors such as pH, temperature, ionic strength, and the presence of cofactors; however, such information is not available in the benchmark dataset introduced by Kroll et al. and therefore cannot be incorporated into the learning process. As a result, both the original model and the proposed approach necessarily learn condition-agnostic representations of enzyme–substrate affinity. Nevertheless, evidence from subsequent datasets, such as MPEK, indicates that enriching models with additional biochemical and contextual information can substantially improve predictive performance, as reflected by the higher Pearson correlation coefficient reported for MPEK (PCC = 0.8398; R^2^ = 0.7049) compared to the original Kroll dataset (PCC = 0.7440; R^2^ = 0.5519). Consistent with observations by Kroll et al., future improvements are expected from the integration of enzyme active-site features (e.g., hydrophobicity, depth, and structural properties) as well as organism-specific physiological parameters, such as typical intracellular pH and temperature, once such annotations become more widely and systematically available.

Despite these promising results, several limitations warrant discussion. Performance dropped when transitioning from the MPEK dataset to Kroll’s dataset, reflecting the ongoing challenge of domain generalization in biochemical prediction. This suggests that future work should focus on transfer learning strategies and multi-dataset training to enhance robustness. Additionally, while our framework captures global features of enzyme–substrate interactions, it does not explicitly model 3D structural information such as solvent accessibility or binding site geometry, which are known to influence Km. Incorporating structural features derived from AlphaFold models or molecular docking simulations could further improve accuracy.

Another important consideration is interpretability. While advanced models such as LSTMs and diffusion regressors offer strong predictive power, they function as “black boxes.” Developing explainable AI methods to identify which sequence motifs or molecular substructures most influence Km predictions will be essential for translating these models into actionable biochemical insights.

## Conclusion

In this work, the developed machine learning framework KmPred achieved a breakthrough in AI-driven enzymology through the precise prediction of Michaelis’ constant Km, using a novel combination of deep protein embeddings and molecular descriptors. This model yielded results that were both robust and generalizable, outperforming existing models with the best performance on the MPEK dataset PCC = 0.8398; R^2^ = 0.7049, which significantly outperformed the baseline MPEK model, and competitive on the Kroll dataset PCC = 0.7440; R^2^ = 0.5519$. This confirmed that it is feasible to predict Km values using computation rather than resource-intensive experimental assays. The predictive models obtained herein are valuable tools for metabolic engineering, systems biology, and drug discovery. Future work will focus on incorporating structural features and exploring transfer learning to improve the generalization of the best models, while developing interpretable AI models in order to extend biological understanding of the enzyme-substrate affinity, thereby accelerating biotechnological innovation in providing strategic creativities, for example, Saudi Vision 2030.

## Data Availability

This study utilized the publicly available Kroll’s and MPEK benchmark datasets for drug–target binding affinity modeling.The complete code base, encompassing data preprocessing, feature generation, model training, and all necessary scripts to reproduce the results presented in this study, the code is made publicly available publicly available at: https://github.com/misharisaud/KmPred to ensure transparency and facilitate future research. The processed data required to reproduce the analyses and figures are publicly available at: https://figshare.com/articles/dataset/KmPred_Dataset/30171538. The original benchmark datasets are publicly distributed by their maintainers and can be accessed as cited in the manuscript. No restrictions apply to the availability of the code and processed data.
